# Analysis of the Complete Genome Sequence of *Cucumber mosaic virus* Strain K

**DOI:** 10.1128/genomeA.00053-18

**Published:** 2018-02-15

**Authors:** Richard Moyle, Lara-Simone Pretorius, Louise S. Shuey, Ekaterina Nowak, Peer M. Schenk

**Affiliations:** a Nexgen Plants Pty. Ltd., School of Agriculture and Food Sciences, The University of Queensland, Brisbane, Queensland, Australia

## Abstract

The complete genome sequence of *Cucumber mosaic virus* strain K was determined by deep RNA sequencing. The tripartite genome consists of a 3,382-nucleotide (nt) RNA1, a 3,050-nt RNA2, and a 2,218-nt RNA3 segment. Phylogenetic analysis placed RNA1 and RNA2 in subgroup IB. However, RNA3 grouped with subgroup IA isolates, indicating a likely recombination event.

## GENOME ANNOUNCEMENT

*Cucumber mosaic virus* (CMV) is the type member of the plant virus genus *Cucumovirus* within the *Bromoviridae* family. CMV is distributed worldwide and is primarily vectored by aphids in a nonpersistent manner (1). CMV has a wide plant host range, infecting more than 1,200 plant species in over 100 families, including vegetables, fruit crops, ornamentals, and weeds (2, 3).

The CMV genome consists of three RNA segments, each individually packaged inside coat protein subunits to form icosahedral particles (4). CMV strains are divided into two major subgroups, I and II, with subgroup I strains further divided into the A and B subgroups (3–5). The CMV K strain (of subgroup 1B) originates from China (6). RNA2 and RNA3 segment sequences were previously published in 1994 and 1999, respectively (5, 7).

Here, we report the complete genome sequences of all three RNA segments of a 2016 version of the CMV K strain, compiled from Illumina platform deep RNA sequencing (RNA-seq) reads. Symptomatic tomato (Moneymaker variety) young leaf tissue was harvested 30 days after mechanical inoculation. Typical CMV symptoms of leaf mottling, shoestrings, and filiformity were evident on new leaf growth from ∼12 days postinoculation. Symptomatic leaf tissue was harvested and pulverized, and total RNA was extracted using previously described methods (8). Deep RNA-seq was performed using Novogene as the service provider.

After adapter trimming, filtering, and subtraction of chloroplast-derived sequences, 24,246,450 clean sequencing reads were obtained. The CMV K 2016 genome was assembled using previously described methods (9–11). To assist assembly, three previously reported sequences (GenBank accession numbers AB179764, S72187, and AF127977) were used as reference genomes for each RNA segment. In total, 5,646,263 reads were assembled, providing full coverage at an average depth of more than 50,000 sequences per nucleotide across all three RNA segments. Similar to prior plant virus genome sequencing reports that used deep RNA-seq (9, 12), CMV K 2016 sequencing revealed that the virus was present as a quasispecies. The CMV K 2016 RNA1 segment was 3,382 nucleotides (nt) in length, and one clear sequence variation (A or G) was identified at nucleotide position 3158. The RNA2 segment was 3,050 nt in length, while the RNA3 segment was 2,218 nt in length and contained clear sequence variations at nucleotide positions 4 and 8 (both A or T).

Alignment of the CMV K 2016 RNA2 segment with the CMV K RNA2 sequence released in 1994 revealed that 35 single nucleotide polymorphisms (SNPs) and three indels were introduced during the 22-year interval in which the isolate was maintained by serial passage. Similarly, alignment to the CMV K RNA3 sequence released in 1999 (5) revealed that 15 SNPs and one indel were introduced during that 17-year interval.

Phylogenetic analysis of all three RNA segments revealed that RNA1 and RNA2 of CMV K 2016 cluster with subgroup IB. However, RNA3 appears to form part of subgroup IA. It is likely the CMV K strain formed after a mixed infection with an isolate from the IA subgroup, resulting in a likely recombination event in RNA3.

### Accession number(s).

The GenBank accession numbers for the CMV K 2016 RNA1, RNA2, and RNA3 sequences have been deposited in GenBank under the accession numbers MG182148 to MG182150.
